# Temperature control and volume measurement in clinical analysers

**DOI:** 10.1155/S1463924688000355

**Published:** 1988

**Authors:** C. Franzini, W. Günther, U. Hagelauer, P. Bonini

**Affiliations:** 1 Ospedale di Rho Corso Europa 250 Milano 20017 Rho Italy; 2 Gebrüder Haake GmbH Karlsruhe Germany; 3 Institute of Biomedical Engineering University of Stuttgart Germany; 4 Istituto Scientifico San Raffaele Via Olgettina 60 Milano 20132 Italy


Journal of Automatic Chemistry Vol. 10, No. 4 (October–December 1988), pp. 171–174

### Temperature control and volume measurement in clinical analysers

**C. Franzini,**
*Ospedale di Rho, Corso Europa 250, 20017 Rho, Milano, Italy*

**W. Günther,**

*Gebrüder Haake GmbH, Karlsruhe, FR Germany*

**U. Hagelauer**
*Institute of Biomedical Engineering, University of Stuttgart, FR Germany*

**and P. Bonini**
*Istituto Scientifico San Raffaele, Via Olgettina 60, 20132 Milano, Italy*

*Temperature control and volume measurement are two instrumental factors that, at different stages of the analytical cycle (both in manual and automatic work), have a significant impact on the quality (accuracy and precision) of the analytical result. This paper reports work carried out by two committees which prepared guidelines on the definition and control of these two instrumental factors of analytical reliability.*

*'Temperature control' is the subject of an 'ad hoc' committee on temperature control (designated AHCTC) set up under the auspices of the Standing Action Committee on Instrumentation (SACI), a technical committee of the European Committee for Clinical Laboratory Standards (ECCLS). The membership of the AHCTC includes C. Franzini (Chairman, Italy), W. Günther (FR Germany), U. Hagelauer (FR Germany) and A. von Klein Wisenberg (FR Germany).*

The AHCTC's starting point was an attempt to define the parameters which describe temperature control and to establish the requirements for such parameters in the clinical laboratory.

The time-course of the temperature in a liquid mass (of temperature *To*), which is transferred in an environment thermostated [1] at a set-point temperature (*Ts*), is shown schematically in figure 1. The AHCTC expressed the opinion [2] that the periodical temperature variation which occurs after the set-point has been approached is better defined in terms of 'permissible deviation [from the set-point]' than in terms of accuracy and precision. The 'equilibration time' and the 'permissible deviation' (figure 1) are the parameters which, together with a third, 'temperature uniformity', describe temperature control in clinical analysis. The latter refers not only to the temperature uniformity among different tubes and/or cuvettes in a rack, in a block or in a rotor, but also to temperature uniformity in a single cuvette (or tube) [3].

The next step was to define the requirements for temperature control, and the permissible deviation was regarded as the most critical parameter for this. Considering that multi-purpose analysers are used to perform different tests, and assuming that the narrower temperature limits are required for enzyme activity determination, the AHCTC attempted to define the permissible temperature deviation for this kind of measurement. Two main criteria were considered [2]—see table 1. Theoretical considerations concerning these two criteria led to the formulation of the following equation:

$$\Delta Tm = \frac{Tm \cdot CVp}{Kt}$$

which relates the permissible deviation ($\Delta Tm$) to the assay temperature ($Tm$), to the partial error due to temperature variation ($CVp$) and to a factor ($Kt$), which is a function of the energy of activation of the enzyme. Assuming, for the most commonly measured enzyme activities, that activation energy values are in the range 35–60 kJ/mol [4], the values for permissible deviation listed in table 2 were calculated.

Following this approach, temperature requirements are not given in fixed terms as previously [5], but in relation

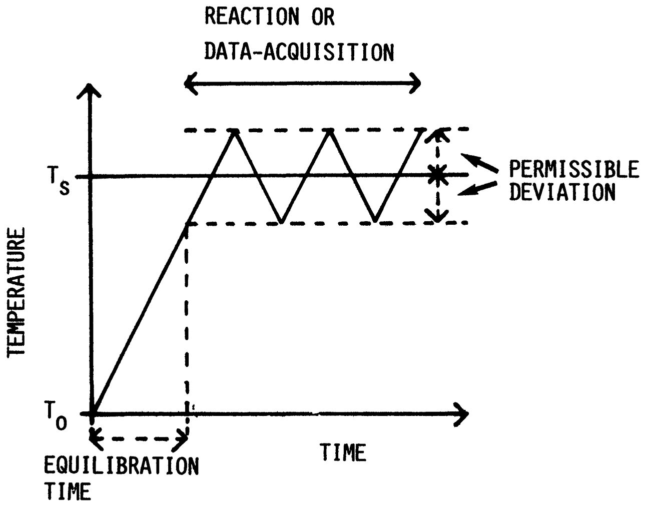

*Figure 1. Time-course of the temperature in a liquid mass at the temperature 'To', transferred in an environment thermostated at the set-point temperature 'Ts'. The equilibration time and the permissible deviation (from the set-point) are the two parameters selected for describing the temperature variation.*

*Table 1. Criteria for defining permissible deviation of temperature in conversion rate measurements.*

| |
|---|
| Influence of the temperature on the reaction rate as a function of the energy of activation of enzyme. |
| Contribution of the 'temperature error' to the overall error of the assay. |

*Table 2. Values for permissible temperature deviation (from the set-point) as a function of the (expected or required) overall error and of the assay temperature.*

| Overall error | Assay temperature | Permissible deviation |
|---|---|---|
| 6% | 30 °C | 0·38 °C |
| 6% | 37 °C | 0·39 °C |
| 4% | 30 °C | 0·25 °C |
| 4% | 37 °C | 0·27 °C |
| 1% | 30 °C | 0·06 °C |
| 1% | 37 °C | 0·07 °C |

to the expected, or desired, overall error in the assay. In practical work, this means that if the other chemical or instrumental factors of the analytical variability (like pH control, volume measurement, absorbance measurement, and wavelength calibration) are not strictly enough controlled to allow for lower than, say, 4% overall error, it may result in a useless effort to achieve better than ±0.2 °K (or ±0.2 °C) temperature control. A temperature control within ±0.05 °K should be aimed at, which will give an overall error in kinetic measurements of 1%. The verification of such a narrow permissible temperature deviation requires a high-quality thermometer, such as a standard platinum resistance thermometer [6]. The high price of these sophisticated systems leads to the question of cost/benefit; one which is difficult to address.

Temperature uniformity should be controlled within the same limits; equilibration time should be short enough to allow the temperature of the incubation mixture ($Ts$) at the start of the kinetic measurement ($ts$) to be:

$$Ts = Tm \pm \Delta Tm.$$

Further work of the AHCTC will involve to the preparation of guidelines to verifying how the expected in-the-cell temperature control is being achieved. Basically, for this operation, appropriate tools and feasible methods for checking calibration are needed. The tools are listed in table 3: the Pt-100 resistance thermometer may represent a 'reference method' [6], but well calibrated thermistors, featuring very low thermal mass and thin enough probes to be introduced in small-volume cuvettes, may be adequate in terms of accuracy and sensitivity. The use of thermochromic solutions [7] may allow temperature verification in fixed or rotating spectrophotometric cuvettes which are unaccessible to the probe, but the inherent accuracy and the sensitivity of this measuring system have to be proved. Other methods, listed in parentheses in table 3, have no practical application at the moment.

*Table 3. Tools for monitoring the temperature in reaction mixtures.*

| |
|---|
| Metal-resistive-temperature-sensors (Pt-100). |
| Semiconductor-resistive-temperature-sensors (thermistors). |
| Thermochromic solutions. |
| (Radiation thermometry) |
| (Liquid crystal thermography) |

The verification tools (thermometers) may be calibrated with reference to the fixed points of the International Practical Temperature Scale-1968 (IPTS-68) or to some suggested fixed points for the life sciences [6], as listed in table 4. Among these, the rubidium triple point standard [8] may be especially useful for checking the accuracy of thermometers intended for measuring temperatures near to 37 °C. In addition to those listed in table 4, the second or third order fixed points (6,9) listed in table 5 may be of help in the range 22.4–42.7 °C. In practical work, although the recommended procedure for calibrating thermistor thermometers includes comparison with a recently calibrated platinum resistance thermometer [10], comparison at three temperature values with a certified mercury-in-glass thermometer, in a well equilibrated water-bath [11], may be an acceptable procedure.

*Table 4. Fixed points for the calibration of thermometers.*

| | |
|---|---|
| *Fixed points of the IPTS (68)* | |
| (Ice point of water): | 0·000 °C |
| Triple point of water: | 0·010 °C |
| Liquid and vapour phases of water: | 100·000 °C |
| *Fixed points for the life sciences (SRMs available from NBS)* | |
| Gallium melting point: | 29·772 °C |
| Rubidium triple point | 39·265 °C |

*Table 5. Calibration or verification of thermometers: additional fixed points in the range 22·36–42·7 °C.*

| | |
|---|---|
| *Second-order fixed points* | |
| Benzoic acid (*tp*): | 22·36 °C |
| Phenoxybenzene (*tp*): | 26·87 °C |
| $Na_2SO_4 \cdot 10H_2O/Na_2SO_4$ (*trsp*): | 32·37 °C |
| n-icosane (*tp*): | 34·49 °C |
| 1,3-dioxolan-2-one (*tp*): | 36·32 °C |
| $KF \cdot 2H_2O/KF$ (*trsp*): | 41·42 °C |
| *Third-order fixed points* | |
| $LiNO_3 \cdot 3H_2O$ (*mp*): | 29·9 °C |
| $CaCl_2 \cdot 6H_20$ (*mp*): | 30·2 °C |
| $Ca(NO_3)_2 \cdot 4H_2O$ (*mp*): | 42·7 °C |

*tp* = triple point; *trsp* = transformation point; and *mp* = melting point.

*Volume measurement is another critical operation in analytical procedures. Problems relating to this operation were considered by the Working Party on Sampling, Diluting and Dispensing (WPSDD), which convened at the suggestion of the Expert Panel on Instrumentation (EPI) of the International Federation of Clinical Chemistry (IFCC). Members of the WPSDD included M. Besozzi (Italy), P. A. Bonini (Italy), G. Caminada (Switzerland), C. Franzini (Convenor, Italy) and G. Luise (Italy). Because of the changed structure of the EPI, the WPSDD ended before any guideline was produced; nevertheless, collaborative efforts produced some results and these are discussed here.*

The first step in the WPSDD's work was to define and classify the volumetric operations relevant to the analy-

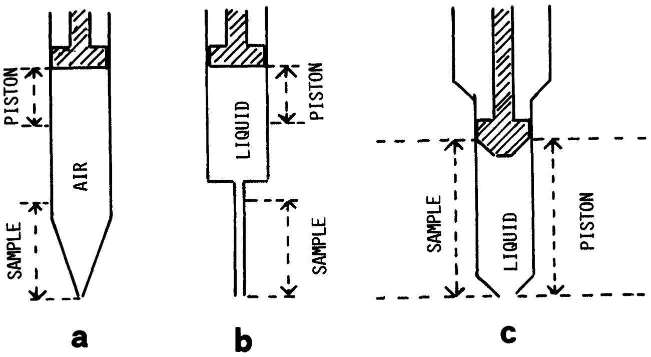

*Figure 2. Three different types of POVA. (a) Air-displacement; (b) liquid-displacement; (c) positive-displacement.*

tical procedures, as shown in table 6. Then, the equipment used to perform the different types of volumetric operations were grouped into three main categories, as shown in table 7. The most commonly used devices, the 'Piston Operated Volumetric Apparatus' (POVA) are described in DIN norms [12], and are also being considered in draft documents by ISO [13].

*Table 6. Volumetric operations relevant to the analytical cycle.*

*Sampling*: A stated volume of the specimen is transferred.
*Diluting: Either* a stated volume of specimen plus a stated volume of reagent is transferred *or* specimen and reagent are transferred in a stated volumetric ratio.
*Dispensing:* A stated volume of reagent is transferred.

*Table 7. Devices for sampling, diluting and dispensing which are commonly used in mechanized analysis.*

(1) Piston-operated-volumetric-apparatus (POVA).
(2) Peristaltic pumps.
(3) Other systems:
Pressure/time operated systems.
Sampling valves.

POVAs can be classified into three different types, as shown schematically in figure 2. In the air-displacement type (figure 2[*a*]), the piston and the fluid being measured move in different zones, and the movement is transmitted from the piston to the liquid by an air cushion. In the liquid-displacement type (figure 2[*b*]) the piston and the liquid again move in different zones, but the movement is transmitted by liquid: this can be either a reagent or a diluent to be added in a stated volume to the measured volume of specimen, or water or an inert fluid. In the positive-displacement type (figure 2[*c*]) the piston and the liquid being measured move in the same zone. The main uses of the different types of POVA in the above mentioned volumetric operations are summarized in table 8.

In automated work, liquid-displacement systems are most commonly used; they can be operated in three main modes (figure 3). The system may be filled with the reagent (or diluent): the desired amounts of reagent and of specimen are aspirated, respectively, from a reservoir

*Table 8. Main uses of the different kinds of POVA.*

| Kind of POVA | Main use |
|---|---|
| Air displacement | Sampling |
| Liquid displacement | Diluting<br>Dispensing |
| Positive displacement | Sampling<br>Dispensing |

and through the tip, and distributed from the tip (figure 3[*a*]). A diluting operation is obtained; if the specimen volume is set to 0, dispensing is achieved. In a second mode (figure 3[*b*]) the system is filled with a washing or inert fluid and both the reagent and the specimen are aspirated and distributed through the tip. Here again diluting and dispensing operations can be performed. These two operating modes include the presence of a valve. POVAs can also be operated in a without-valve mode, as shown in figure 3(*c*). This last operating mode also allows sampling operations to be easily performed.

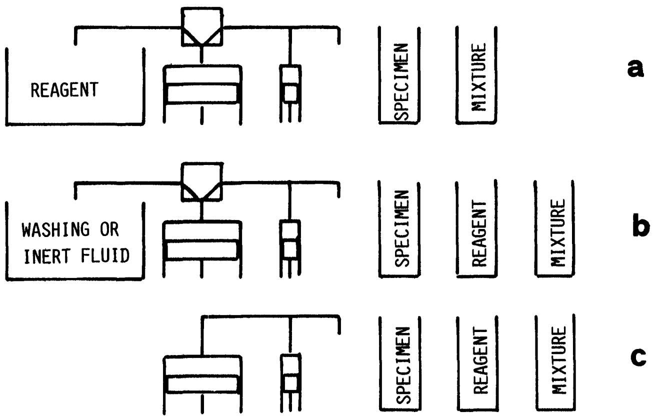

*Figure 3. Three main different modes for operating liquid-displacement POVAs. (a) and (b) With-valve operation; (c) without-valve operation.*

Since the reagent-to-sample volume ratio is frequently high, the systems operate with two pistons (syringes) of different diameter, but single-piston instruments may also be operated in three modes cited above.

POVAs' pistons are driven in different ways, see table 9. Significant improvements have recently been achieved in this by the introduction of microprocessor-controlled stepper motor driven pistons; the additional monitoring, via external sensors, of the fluid's level may lead to further reliability of volume measurements in the 1 μl range.

*Table 9. Operation of the piston in POVAs.*

Manual, with mechanical stops.
Pneumatic, with mechanical stops and valves.
Motor-driven, mechanical or electrical stops.
Microprocessor-controlled stepped motor.
Microprocessor-controlled stepped motor with additional monitoring (sensors) of the fluid's level.

The performance characteristics of volumetric apparatus may be defined by precision and accuracy of volume

*Table 10. Methods for the verification of the performance of volumetric apparatus.*

| |
|---|
| 'Primary' method of verification: gravimetry. |
| 'Secondary' method of verification: spectrophotometry of dye solutions, in comparison with gravimetrically checked volumetric pipettes and flasks. |

measurements, and by carry-over and dead volume. These characteristics may be verified by means of 'primary' and 'secondary' methods [14], as shown in table 10. Since the secondary methods are based upon comparison with gravimetrically verified volumetric equipment, the assessment ultimately relies upon gravimetry. Specifications concerning balance performance, and calibration by means of NBS standards, procedures and statistical calculations have been published [14]. In the secondary methods, the use of potassium dichromate [14] and of Evans blue [15] solutions has been suggested. For the secondary-method verification of samplers intended for serum measurement, the use of iso-viscosity dye solution, such as Evans-blue-dyed-serum, has been suggested [16]: this may be particularly important for the verification of air-displacement POVAs.

### Acknowledgements

The collaboration of the members of WPSDD, M. Besozzi (Ospedale Del Ponte, Varese, Italy), G. Caminada (Hamilton Bonaduz AG, Bonaduz, Switzerland), and G. Luise (Instrumentation Laboratory SpA, Milano, Italy) is gratefully acknowledged; and the frequent advice of A. von Klein Wisenberg (Instand, Freiburg, FR Germany), a member of AHCTC, was of great help.

### References

1. IFCC-EPI. The effect of instrumental and environmental factors on the thermal regulation of the temperature of incubation. *IFCC Document, Stage 2, Draft 1* (1985).
2. ECCLS. *Guidelines for Temperature Control in Clinical Chemistry. Part. I: Requirements for temperature control. First Draft* (1978).
3. Hagelauer, U., Arnadov, K. and Faust, U., *International Laboratory* (June 1986), 62.
4. Lentner, C. (Ed), *Tavole Scientifiche Geigy*, 8th edn (Edizione italiana). Ciba-Geigy Ltd (Basel, 1984).
5. IFCC-EPE, *Approved recommendation (1978) on IFCC methods for the measurement of catalytic concentration of enzymes.* Reproduced in *Clinica Chimica Acta*, **98** (1979), 165F.
6. Shooley, J. F., *Thermometry* (CRC Press, 1986).
7. O'Leary, T. D., Badenoch, J. L. and Bais, R. *Annals of Clinical Biochemistry*, **20** (1983), 153.
8. Mangum, B. W., A temperature reference standard near 39.30 °*C. NBS Special Publication 260–87* (1983).
9. von Klein Wisenberg, A., Personal communication (1987).
10. NCCLS, *Guide for the Selection of Accuracy Class of Thermistor Thermometers and the Verification of Their Accuracy* (NCCLS, 1979).
11. Ween, S., *International Clinical Products Review*, **4** (1985), 65.
12. DIN, *Volumenmessgerate mit Hubkolben. DIN 12650, Teil 1* (1978), *Teil 2* (1981), *Teil 3* (1981), *Teil 4* (1981), *Teil 5* (1981).
13. ISO, *Piston and/or Plunger Operated Volumetric Apparatus (POVA). Part 1: Definitions; Part 2: Operating considerations: Part 3: Test methods; Part 4: Specifications (Drafted documents).*
14. NCCLS, *Determining Performances of Volumetric Equipment. Proposed Guideline.* Vol. 4, No. 6 (1984).
15. Geary, T. D. and Farrance, I., *Clinical Biochemistry Reviews*, **3** (1981).
16. Duncan, I. W., Mather, A. and Cooper, G. R., *The Procedure for the Proposed Cholesterol Reference Method* (Center for Disease Control, Atlanta, Georgia, 1982).

**DIODE-ARRAY SPECTROSCOPY**

For scientists learning about or using UV/visible spectrophotometers, Hewlett-Packard Company has published a gratis 64-page book by Dr A. J. Owen, HP scientist, that describes the advantages of diode-array over conventional mechanical-scanning technology in many applications. For teaching purposes, there are sections that outline the history and theory of UV/visible spectrophotometric techniques.

Detailed explanations of how diode-array spectrophotometers work and how to get the best results for different applications provide a clear picture of when to use a particular capability.

The book fully explains the advantages of diode-array technology, including faster, more accurate and precise results; higher productivity; greater reliability; and lower cost of ownership.

*Copies from any local HP Sales Office or Hewlett-Packard S.A., Route du Nant-d'Avril 150, PO Box, CH-1217 Meyrin 2, Geneva, Switzerland.*

